# *KRN4* Controls Quantitative Variation in Maize Kernel Row Number

**DOI:** 10.1371/journal.pgen.1005670

**Published:** 2015-11-17

**Authors:** Lei Liu, Yanfang Du, Xiaomeng Shen, Manfei Li, Wei Sun, Juan Huang, Zhijie Liu, Yongsheng Tao, Yonglian Zheng, Jianbing Yan, Zuxin Zhang

**Affiliations:** 1 National Key Lab of Crop Genetic Improvement, Huazhong Agricultural University, Wuhan Hubei, People's Republic of China; 2 College of Agronomy, Hebei Agricultural University, Baoding Hebei, People's Republic of China; 3 Hubei Collaborative Innovation Center for Grain Crops, Jingzhou Hubei, People's Republic of China; University of Minnesota, UNITED STATES

## Abstract

Kernel row number (KRN) is an important component of yield during the domestication and improvement of maize and controlled by quantitative trait loci (QTL). Here, we fine-mapped a major KRN QTL, *KRN4*, which can enhance grain productivity by increasing KRN per ear. We found that a ~3-Kb intergenic region about 60 Kb downstream from the SBP-box gene *Unbranched3* (*UB3*) was responsible for quantitative variation in KRN by regulating the level of *UB3* expression. Within the 3-Kb region, the 1.2-Kb Presence-Absence variant was found to be strongly associated with quantitative variation in KRN in diverse maize inbred lines, and our results suggest that this 1.2-Kb transposon-containing insertion is likely responsible for increased KRN. A previously identified A/G SNP (S35, also known as Ser220Asn) in *UB3* was also found to be significantly associated with KRN in our association-mapping panel. Although no visible genetic effect of S35 alone could be detected in our linkage mapping population, it was found to genetically interact with the 1.2-Kb PAV to modulate KRN. The *KRN4* was under strong selection during maize domestication and the favorable allele for the 1.2-Kb PAV and S35 has been significantly enriched in modern maize improvement process. The favorable haplotype (Hap1) of 1.2-Kb-PAV-S35 was selected during temperate maize improvement, but is still rare in tropical and subtropical maize germplasm. The dissection of the *KRN4* locus improves our understanding of the genetic basis of quantitative variation in complex traits in maize.

## Author Summary

Maize (*Zea mays* L.) is one of the world's most important sources of calories for humans. With an expanding global population, the demands for maize-derived food, feed, and fuel are rapidly increasing. To meet these needs, geneticists and breeders are facing the challenge of enhancing grain yield through genetic improvement of maize germplasm. Understanding the genetic basis of grain yield is necessary to guide breeding efforts towards the development of high-yielding hybrids. Kernel row number (KRN) in maize is one of the most important yield components and a significant breeding target. Over the last few decades, many genes that determine inflorescence development and architecture have been identified and characterized. The formation of kernel rows is an integral part of the development of the female inflorescence in maize. Nevertheless, the genetic basis and molecular regulation of quantitative variation in KRN is poorly understood. This study provides experimental evidence for the hypothesis that variation in intergenic regions can regulate quantitative variation of important grain yield-related traits, and also provides tools for improving KRN in maize.

## Introduction

Understanding the genetic and molecular basis of grain yield is necessary to guide breeding efforts towards the development of high-yielding maize hybrids. Kernel row number (KRN) in maize is one of the most important yield components and a significant breeding target. During the domestication of maize, KRN underwent a dramatic change from two rows in teosinte to more than eight rows in modern maize [1]. A number of quantitative trait loci (QTL) have been reported [2–3] to control quantitative variation in KRN. However, the genetic and molecular mechanisms of these KRN QTL are unknown.

Switching from vegetative to reproductive development turns axillary meristems (AMs) into ear inflorescence meristems (IMs) [4]. The IMs then elongate and produce spikelet-pair meristems (SPMs). Each SPM makes two spikelet meristems (SMs), which then give rise to floral meristems (FMs) that form kernels after fertilization [4]. The initial number of SPMs on the female inflorescence meristem determines the number of kernel rows on the maize ear, while the meristematic activity of IMs determines the potential number of kernels in each kernel row. The initial number of SPMs is correlated with the size of the inflorescence meristem, which provides space for the development of SPMs. The *CLAVATA*-*WUSCHEL* (*CLV*-*WUS*) feedback-signalling loop regulates IM size by restricting stem cell proliferation and maintaining meristem activity. Recently, several genes in the *CLV*-*WUS* feedback loop, including *thick tassel dwarf1* (*td1*) [5], *fasciated ear2* (*fea2*) [6–7], and *COMPACT PLANT2* (*CT2*) [8], were isolated in maize. Additionally, the *RAMOSA* genes [9], *Corngrass1* (*Cg1*) [10], *tasselsheath4* (*tsh4*) [11], *FLORICAULA/LEAFY* (*ZFL1* and *ZFL2*) [12], *unbranched2* (*ub2*) and *ub3* [13] and others, all affect ear morphology by regulating the development of SPMs and SMs. However, these genes were originally isolated through genetic assays of inflorescence mutants, the mechanisms of them to affect quantitative variation of ear-related traits remain unknown, except for *fea2* and *ub3* [7, 13]. Thus, the genetic basis and molecular regulation of quantitative variation in KRN deserves further study.

Previously, a major KRN QTL, *KRN4*, with a large additive effect was identified by combining linkage and association mapping [2–3]. We found that the associated SNPs within *KRN4* constitute a linkage disequilibrium block (Chr4:198.9Mb–199.9Mb) in our association mapping panel (S1 Fig). In the present study, we isolated *KRN4* by positional cloning and analysed the putative causal variant using maize mutants, gene expression, and association mapping. We then examined changes in the allelic composition of populations for the causal variant during the domestication and improvement of maize. Finally, we assessed the utility of *KRN4* for maize breeding by allele substitution using marker-assisted selection.

## Results

### Positional cloning of *KRN4*

**Fig 1:**
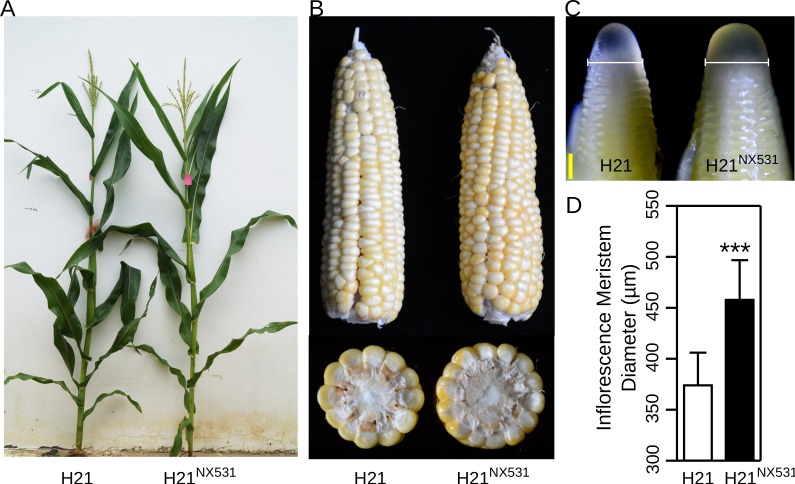
The plant and inflorescence performance of H21 and H21^NX531^. A) The plant performance of H21 (right) and H21^NX531^ (left). B) The mature ears of H21 and H21^NX531^. C) Diameter of inflorescence meristems in H21 and H21^NX531^, micrograph of apical 2-mm immature ears of H21 (left) and H21^NX531^ (right), white bracketed lines represent inflorescence meristem diameters. Bar = 200 μm. D) The statistical analysis of inflorescence meristem diameters between H21 and H21^NX531^. *** *P* < 0.001. N: 8/7 for H21 and H21^NX531^.

**Fig 2:**
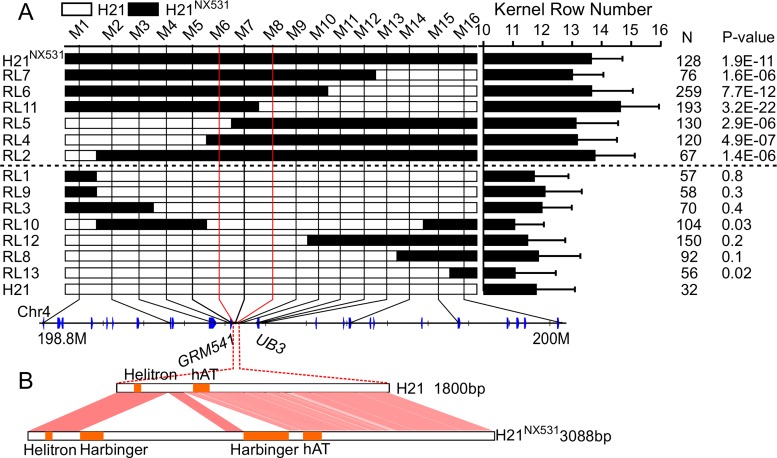
Fine mapping of *KRN4*. A) The graphical genotypes of homozygous recombinants (HR). The white boxes in the graphical genotype represent the genomic segments from H21, the black boxes represent genomic segments from H21^NX531^. A progeny test was conducted to examine whether the KRN of HRs were significantly higher than that of H21. N: the total number of HR phenotyped in four environments (S1 Dataset). *P*-value: Student’s t-test of the difference in KRN between HRs and H21. The axis represents the physical map of *KRN4*, and the blue box represents genes within *KRN4*. B) Nucleotide sequence differences in the *KRN4* region between H21 and H21^NX531^. The orange solid boxes represent the locations of transposable elements in *KRN4*.The shadowed regions represent homologous *KRN4* sequence between H21 and H21^NX531^ (S2 Dataset).

To fine-map *KRN4*, a near isogenic line (H21^NX531^) containing the QTL was developed. In comparison with H21, H21^NX531^ exhibited similar plant appearance (Fig 1A). The KRN (*P*-value = 5.87 E^-07^), ear diameter (*P*-value = 0.0017), cob diameter (*P*-value = 0.0075), kernel number (*P*-value = 8.70 E^-05^), and grain yield (*P*-value = 7.47 E^-05^) were significantly increased in H21^NX531^ (Table 1 and Fig 1B). However, 100-kernel weight of H21^NX531^ did not differ from that of H21 (Table 1). To understand the developmental basis of the increase in KRN, we measured the inflorescence meristem size of the 2-mm immature ear. The diameter of ear IM in H21^NX531^ is significantly larger (*P*-value = 5.2 E^-04^) than that of H21 in the developing female inflorescence (Fig 1C and 1D). Next, to fine map *KRN4*, a total of 31 recombinants representing 13 distinct crossover events were found in over 10,000 F2 individuals derived from the cross H21×H21^NX531^. We compared the KRN of H21 with homozygous recombinant lines derived from the 13 representative recombinants, and found that the homozygous recombinant lines (RL2, RL4, RL5, RL6, RL7, and RL11) carrying the H21^NX531^ genomic segment between marker M6 and M8 displayed higher KRN (more than 13 rows, *P*-value < 1.0 E^-05^, Student’s t-test) than H21 (11.8 ± 1.3), while the other homozygous recombinant lines carrying the H21 genomic segment exhibited almost the same KRN as H21 (Fig 2A). To exclude the effect of residual genetic background, we also compared the KRN of offspring individuals derived from each of the 13 heterozygous recombinants in four environments. We found that only when the offspring populations were segregated with *KRN4* ^*H21*^ and *KRN4* ^*NX531*^ in M6-M8 marker interval (RL6-RL10, S1 Dataset), the KRN of those individuals with the homozygous H21^NX531^ genotype in the M6-M8 marker interval were significantly higher than that of individuals with the homozygous H21 genotype (*P*-value < 0.01, S1 Dataset, Student’s t-test). Therefore, we could narrow the genomic location of *KRN4* down to a 3-Kb intergenic region flanked by M6 and M8 markers (Fig 2A and S1 Dataset), which is located ~60 Kb downstream from an SBP-box gene *UB3* [13] and ~300 bp upstream of a gene of unknown function, GRMZM2G001541 (Fig 2A). The genomic region between marker M6 and M8 was defined as *KRN4*. In comparison with H21, two regions totaling 1.2 Kb in length (the 1.2-Kb PAV) each containing a fragment of the *harbinger* transposable element are present in H21^NX531^ (Fig 2B and S2 Dataset). Several SNPs and small indels are also present in this region (Fig 2B and S2 Dataset). Therefore, sequence differences within the 3-Kb genomic region between H21 and H21^NX531^ could be the potential causative sites for *KRN4* to control KRN variation.

**Table 1:** Pleiotropic effects of *KRN4*.

Trait	H21	H21^NX531^	P-value	N^a^
Kernel row number	11.1 ± 1.2	13.3 ± 1.2	5.85 E^-07^	17/24
Ear diameter (cm)	4 ± 0.3	4.4 ± 0.3	0.0017	17/24
Cob diameter (mm)	29 ± 1.5	30.8 ± 2.5	0.0075	17/24
Kernel number per ear	309.6 ± 41.7	366.3 ± 32.5	8.70 E^-05^	17/24
Kernel yield per ear (g)	64.8 ± 11.1	81.1 ± 10.4	7.47 E^-05^	15/23
Tassel branch number	8.7 ± 3.6	10.2 ± 4	0.21	17/24
Ear length (cm)	12.5 ± 1.3	11.8 ± 1.2	0.07	17/24
Kernel number per row	28.2 ± 2.6	27.4 ± 2.6	0.31	17/24
100-kernel weight (g)	12.4 ± 1.5	12.8 ± 1.6	0.49	17/24

^a^N, sample size, H21/H21^NX531^

### Expression analysis of *UB3* and *GRMZM2G001541*

**Fig 3:**
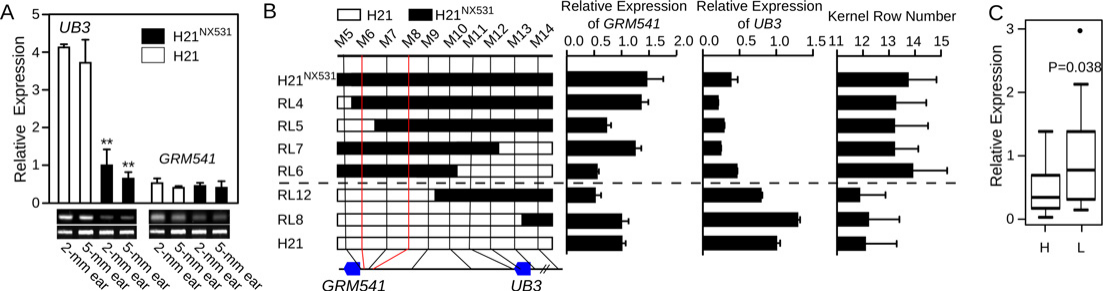
Analysis of *UB3* and GRMZM2G001541 expression. A) Expression patterns of *UB3* and GRMZM2G001541 (*GRM541*) in immature ears of H21 and H21^NX531^, **: *P*-value < 0.01. B) Analysis of *UB3* and GRMZM2G001541 (*GRM541*) expression in recombinant lines in immature 2-mm ear. The white boxes in the graphical genotype represent the genomic segment from H21, and the black boxes represent the genomic segment from H21^NX531^. C) Expression of *UB3* in immature 2-mm ear in 38 diverse inbred lines. H represents lines with the H21^NX531^genotype at the *KRN4* locus (N = 12), and L represents lines with the H21genotype at the *KRN4* locus (N = 26).

We first examined the expression atlas for *UB3* and GRMZM2G001541. The expression data were obtained from qteller (http://www.qteller.com/) and MaizeGDB (http://www.maizegdb.org/). We found both *UB3* and GRMZM2G001541 exhibited similar mRNA expression patterns and accumulated in developing ears and tassels (S2 Fig). They also express in the non-reproductive tissues such as leaf, internode etc. (S2 Fig). However, in the immature ear at spikelet-pair meristems (2-mm ear) and spikelet meristems (5-mm ear) differentiation stages, only *UB3* exhibited differential expression between H21 and H21^NX531^, with an expression level almost threefold higher in H21 than in H21^NX531^ (Fig 3A). Differential expression of *UB3* was also observed in stems, roots, and leaves (S3A Fig). However, in 5-mm tassel and 10-mm tassel, expression of *UB3* did not show an obvious decrease in H21^NX531^ relative to H21 (S3A Fig), which might explain why tassel branch number did not differ between H21 and H21^NX531^ (Table 1). To explore the relationship between expression of *UB3* and *KRN4*, we analysed the expression of *UB3* and GRMZM2G001541 in immature ears of six homozygous recombinant lines (RL4, RL5, RL6, RL7, RL8, and RL12) and two parental lines (H21 and H21^NX531^), and found that RL4, RL5, RL6, and RL7, which carry the *KRN4* ^*NX531*^ allele, showed lower expression of *UB3* and higher KRN, while the lines RL8 and RL12, which carry the *KRN4* ^*H21*^ allele, showed higher expression of *UB3* and correspondingly lower KRN (Fig 3B). In contrast, the expression of *GRMZM2G001541* in the lines with the *KRN4* ^*NX531*^ allele was similar to that in lines with the *KRN4* ^*H21*^ allele (*P*-value = 0.42) (Fig 3B). Therefore, the expression of *UB3* is regulated by *KRN4*, shows a strong negative correlation with KRN (Fig 3B). We further divided these 38 diverse maize inbred lines into two groups: Group L carrying the *KRN4* ^*H21*^ allele (N = 26) and Group H carrying the *KRN4* ^*NX531*^ allele (N = 12), according to their genotypes for the 1.2-Kb PAV of *KRN4* (S1 Table). By examining *UB3* expression at the 2-mm ear stage, we found that the expression of *UB3* in Group L lines was significantly higher than that in Group H lines (*P*-value = 0.038, Student’s t-test, Fig 3C), and KRN in these 38 inbred lines was again negatively correlated with the expression level of *UB3* (*r* = -0.35, *P*-value = 0.037, Pearson’s correlation coefficient, S3B Fig).

### DNA sequence variation and putative causal polymorphic sites in *KRN4* and *UB3*

We sequenced *KRN4* (~3 Kb, between marker M6 and M8) and *UB3* genic region (~4 Kb, including promoter to 3′-UTR but not first intron) in our association mapping panel (S3 Dataset) [3, 14], and identified 69 and 46 polymorphic sites, respectively, with Minor Allele Frequency (MAF) ≥ 0.05 (S4 Fig). Association analysis using the MLM K + Q model [15–16] revealed that four sites were associated with KRN at *P*-value <1.0 E^-04^ (Table 2), including one A/G SNP in the third exon of *UB3* (S35, *P* = 3.81E^-08^, N = 428), one G/A SNP in the 3'-UTR region of *UB3* (S45, *P*-value = 7.35 E^-05^, N = 384), one ~700 bp insertion/deletion (S23, *P*-value = 6.69 E^-05^, N = 416) in the promoter region of *UB3*, and the 1.2-Kb PAV in *KRN4* (*P*-value = 7.28 E^-06^, N = 428) (Table 2). The four sites could be classified into three LD groups at R^2^ > 0.4: group 1 including S23, group 2 including S35 and S45, and group 3 including the 1.2-Kb PAV (S4 Fig). Conditional association analysis was then conducted using these four sites as covariates under an MLM K + Q model, to determine whether these sites were independent or not. When S35 was conditioned, neither S45 nor S23 were significantly associated with KRN (*P*-value 0.49 and 0.41, S2 Table), but the 1.2-Kb PAV was found to be weakly associated with KRN (*P*-value = 0.03, S2 Table). The signals for association of S35 and the 1.2-Kb PAV with KRN were only slightly decreased when conditioned by any one of S23 and S45 (S2 Table). Finally, when conditioned on the 1.2-Kb PAV, the other variants were also still significantly associated with KRN (S2 Table). Hence, the association of the 1.2-Kb PAV with KRN might be independent of S23 and S45 but partially related to S35, and the association of S23 and S45 with KRN might depend on that of S35. The dependence of S45 on S35 might be due to its high linkage disequilibrium with S35; thus, S35 could actually represent the association of S45 with KRN, while S23 might not, because of the weak linkage disequilibrium between S23 and S35 (R^2^ = 0.21).

**Table 2:** The four polymorphisms in *KRN4* and *UB3* associated with KRN under the MLM K + Q model.

Site	Location	Allelea	Frequency^a^	P-value
S23	Promoter of *UB3*	700/170/0-bp Insertion	86/86/243	6.69 E^-05^
S35	Exon of *UB3*	A/G	59/369	3.81 E^-08^
S45	3' UTR of *UB3*	G/A	104/280	7.35 E^-05^
1.2-Kb PAV	*KRN4*	1.2-Kb Presence/Absence	153/257	7.28 E^-06^

^a^ The alleles represent ‘desirable allele/undesirable allele’.

**Fig 4:**
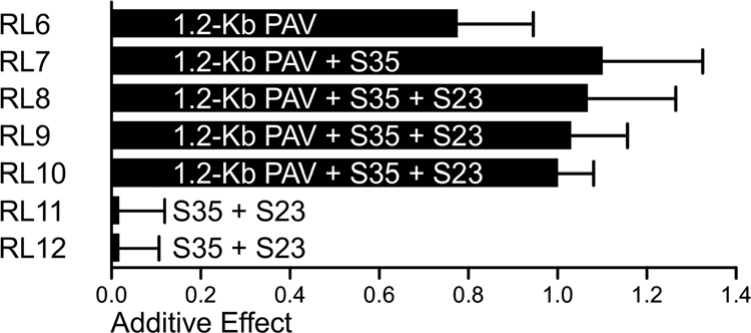
The additive effects of 1.2-Kb PAV and S35 estimated in RLs. The heterozygous recombination lines RL6-RL12 were selfed to generate segregating populations for either 1.2-Kb PAV, S35, and S23, or all of them. The average additive effects estimated in four environments for each RL were treated as the genetic effect of the segregating site in the RL.

To further determine the relationship between the 1.2-Kb PAV, S35, and S23, the segregating populations derived from selfing the heterozygous recombinants RL6-RL12 were used to evaluate the additive effects of these three tightly linked loci. The 1.2-Kb PAV showed a large additive effect (0.78) in RL6 offspring segregating population, while the additive effect of S35 and S23 were zero in RL11-RL12 (Fig 4). However, combination of the 1.2-Kb PAV + S35 (RL7) or the 1.2-Kb PAV + S35 + S23 (RL8-RL10) had an additive effect more than 1.07 rows, almost 40% higher than that of the 1.2-Kb PAV alone in RL6 (Fig 4). These two kinds of combinations exhibited a similar additive effect, which suggests that the increased additive effect was caused mainly by S35 or polymorphisms tagged by S35. Therefore, the 1.2-Kb PAV or a locus near 1.2-Kb PAV that genetically interacts with a locus tagged by S35, and their interaction, might strongly promote the additive effect on KRN (Fig 4). We next constructed haplotypes using 1.2-Kb PAV and S35 (1.2-Kb-PAV-S35) and found that they showed stronger association with KRN (*P*-value = 2.41 E^-09^, N = 428, MLM K + Q) than did each individual locus, when comparing the high-KRN haplotype against the low-KRN haplotype using the MLM K + Q model. In the association mapping panel, a total of four haplotypes (Hap1-Hap4) were observed for the 1.2-Kb-PAV-S35 (Table 3). Lines with Hap1 exhibited higher KRN than lines with the other three haplotypes, and lines containing Hap2 to Hap4 did not significantly differ from each other in KRN (Table 3).

**Table 3:** KRN and frequencies of haplotypes between 1.2-Kb PAV and S35.

Haplotype	1.2-Kb PAV	S35	KRN	Frequency in Maize		
				All Maize^a^	TST^b^	TEMP^c^
Hap1	1.2-Kb Presence	A	14.5 ± 1.9	12.8%	2.5%	21.1%
Hap2	1.2-Kb Presence	G	13.2 ± 1.3	23.3%	36.5%	12.6%
Hap3	1.2-Kb Absence	A	13.4 ± 0.5	0.4%	0.0%	0.8%
Hap4	1.2-Kb Absence	G	13.0 ± 1.5	63.5%	61.0%	65.4%

^a^ The sample size for all maize is 428

^b^ TST: Tropical and SubTropical maize germplasm, sample size: 234

^c^ TEMP: Temperate maize germplasm, sample size: 194.

### Analysis of the molecular evolution of *KRN4*

**Fig 5:**
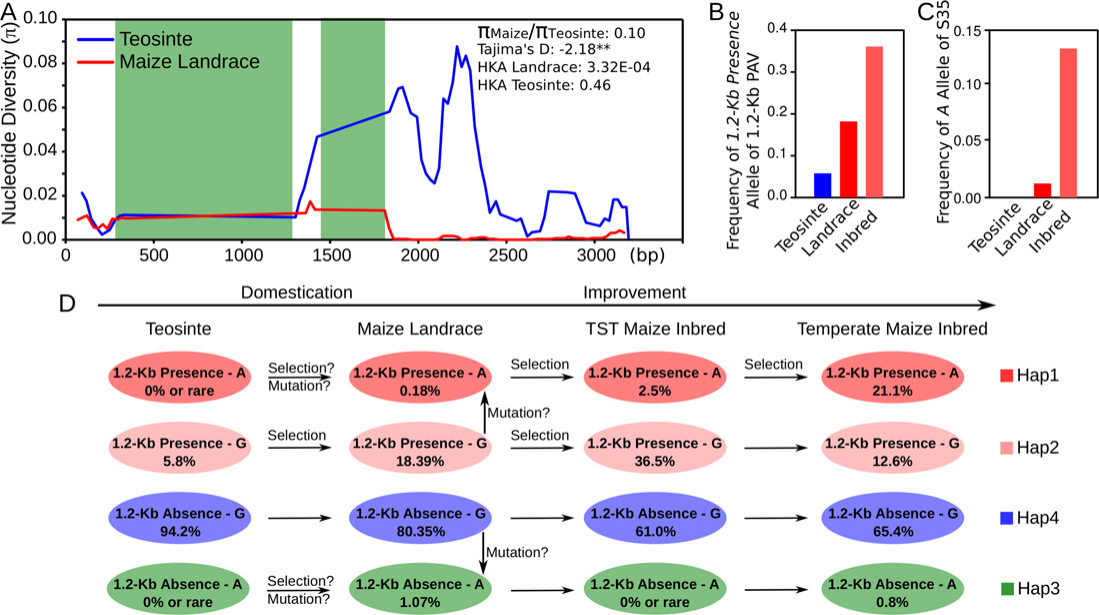
The evidence of significant selection in *KRN4* during maize domestication and improvement. A) Nucleotide diversities (π) within ~3 Kb of *KRN4* for maize landrace (red line) and teosinte (blue line). The green shades represent the 1.2-Kb PAV region. **: *P*-value < 0.01. B) Allele frequencies of *1*.*2-Kb Presence* of 1.2-Kb PAV in teosinte, maize landrace and inbred lines. C) Allele frequencies of *A* of S35 in teoisnte, maize landrace and inbred lines. D) A putative evolutionary pattern of 1.2-Kb PAV and S35 during maize domestication and improvement. The circles in colors represent the four haplotypes between 1.2-Kb PAV and S35. The genotypes of haplotypes in 1.2-Kb PAV and S35 are showed in the circles. The number inside the circle is the frequency of the haplotype. TST maize: Tropical and SubTropical maize germplasms. All of the steps marked as “Selection” are of significantly frequency change, *P*-value < 0.001 (χ^2^ test based on the frequencies).

A total of 29 maize wild relatives *Z*. *mays* subsp. *parviglumis* teosinte accessions and 36 diverse maize landraces were employed to estimate the selection pressure during maize domestication (S4 Dataset). The genomic sequence of *KRN4* was sequenced in them. Then three expectations of past selection were assessed. First, we compared the nucleotide diversity (π) of *KRN4* between teosintes and maize landraces. We found *KRN4* had undergone strong reduction in nucleotide diversity from teosintes to maize landraces with π_maize_/π_teosinte_ = 0.10, indicating that only 10% nucleotide diversity in teosintes was retained in maize landraces (Fig 5A). Second, a significantly negative Tajima’s D-statistic (-2.18, *P*-value < 0.01, length of tested region = 3,144 bp, number of sites = 1,722, Fig 5A) of *KRN4* was acquired in maize landraces which suggested a recent selection in the *KRN4* region. Furthermore, the Hudson–Kreitman–Aguade (HKA) test was applied to assesses the ratio of diversity in maize landrace to divergence from an outgroup (*Z*. *diploperennis*) for *KRN4* relative to four neutral genes. *KRN4* in landrace showed significant selection based on HKA test result (*P*-value = 3.32E^-04^, length of tested region = 3,144 bp, number of sites = 1,722, Fig 5A and S3 Table), but *KRN4* in teosinte doesn’t (*P*-value = 0.46, length of tested region = 3,300 bp, number of sites = 1,642, Fig 5A and S3 Table). These results revealed that *KRN4* was under strong selection during domestication from teosinte to maize, similar to *tga1* promoter and *tb1*upstream region [17–18]. However, different from *tga1* and *tb1* loci [17–18], no fixed difference between teosintes and maize landraces could be observed in *KRN4*.

To explore the evolution of 1.2-Kb-PAV and S35 loci, we genotyped them in 120 teosinte accessions, 280 maize landraces (S5 Dataset) and 428 maize inbred lines, respectively. In teosinte, the frequencies of favorable alleles for 1.2-Kb PAV (*1*.*2-Kb Presence* allele) and S35 (*A* allele) were 5.8% and 0% (Fig 5B and 5C). The *1*.*2-Kb Presence* allele had a higher frequency (9.6%) in *Z*. *parviglumis* but rare in *Z*. *mexicana* (2.4%), implying that the favorable allele of 1.2-Kb PAV in modern maize was probably selected from *Z*. *parviglumis* (S5 Dataset). In maize landrace, the frequencies of favorable alleles for 1.2-Kb PAV and S35 were increased to 18.6% and 1.25%, respectively (Fig 5B and 5C). During modern maize improvement, they were enriched to 36.1% and 13.2% (Fig 5B and 5C), and the R^2^ of them in the association mapping panel were 5.0% and 12.2%. The favorable haplotype of 1.2-Kb-PAV-S35, Hap1 was not detected in teosinte accessions (Fig 5D), and the frequency of Hap1 in maize inbred lines increased to 12.8% (N = 428, Fig 5D), but differed dramatically between temperate (21.1%, N = 234, Fig 5D) and TST (tropical and subtropical, 2.5%, N = 194, Fig 5D) maize inbred lines. The unequal distribution of Hap1 in different subpopulations suggests that favorable Hap1 has been selected to increase grain yields by increasing the number of kernel rows in temperate germplasm. Based on these results, we proposed an evolutionary pattern of 1.2-Kb PAV and S35 during maize domestication and improvement (Fig 5D). Hap2 of 1.2-Kb-PAV-S35, which harbors the *1*.*2-Kb Presence* allele, was selected and enriched from teosinte to landrace and then to tropical and subtropical maize inbred lines (Fig 5D). The favorable Hap1 allele might have been selected from teosinte or could have arisen by mutation at S35 after domestication (Fig 5D). However, the intensive selection on Hap1 only occurred during temperate maize inbred lines improvement (Fig 5D).

### *UB3* regulates inflorescence meristem development

**Fig 6:**
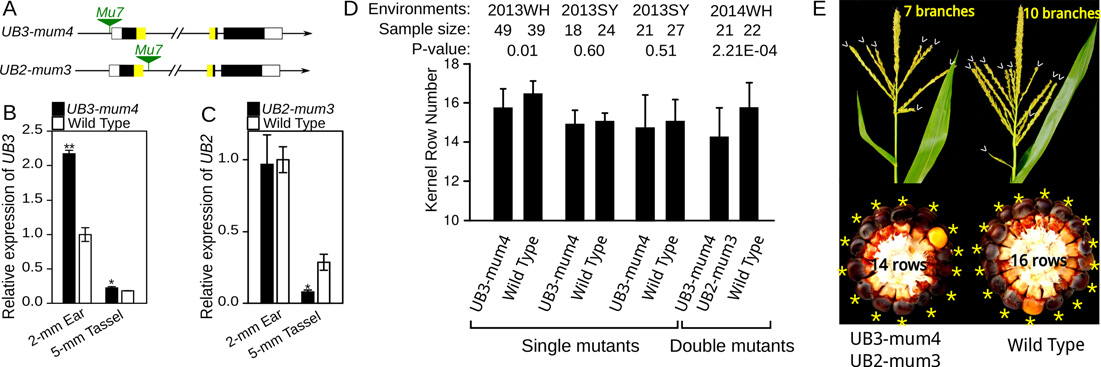
Expression analysis and phenotypic characterization of *UB3-mum4* and *UB2-mum3* mutants. A) The insertion site of *Mutator* in *UB3-mum4* and *UB2-mum3* mutants. B and C) Expression level of *UB3* (B) and *UB2* (C) in mutant and wild type. ** P < 0.01, * P < 0.05. D) KRN performance single and double mutants of *UB3-mum4*/*UB2-mum3* and wild type; WH: Wuhan; SY: Sanya. E) Tassel and ear of wild type and double mutant of *UB3-mum4*/*UB2-mum3*. Detailed information regarding phenotypes is presented in S3–S6 Tables.

*UB3* is an ortholog of *OsSPL14*, which is responsible for *IPA1* (*ideal plant architecture 1*) and *WFP* (*WEALTHY FARMER’S PANICLE*) in rice (S5 Fig) [19–20], and is also homologous with *UB2*. Recent study has revealed that *ub2* and *ub3* knock-out mutants exhibit increase in maize KRN [13]. Two novel *Mutator*-mediated mutants, *UB3-mum4*, with a *Mu7* insertion in the promoter region of *UB3*, and *UB2-mum3*, with a *Mu7* insertion in the first intron of *UB2* (Fig 6A), were obtained from Maize Stock Center. *UB3* expression in 2-mm immature ears and 5-mm tassels of the *UB3-mum4* line was significantly higher than that in the wild type (WT) (Fig 6B). Similarly, a previous study has identified that a *Mu* transposon insertion in 5’UTR of *P1* gene increases *P1* expression in maize [21]. *UB2* expression in 2-mm immature ears of the *UB2-mum3* line did not differ significantly from WT (Fig 6C), but ~14% of *UB2-mum3* transcripts contained an extra 295-bp fragment composed of a 145-bp intron sequence flanking *Mu7* insertion sites and a 150-bp terminal inverted repeat of *Mu7* (S6 Fig). The 295-bp fragment was inserted into the SBP-box domain-encoding sequence and might result in loss of function of the alternatively spliced transcript. We developed segregating populations to evaluate the influence in KRN by the *Mu7* insertion in *UB3-mum4* and *UB2-mum3*. Each single mutant did not show an obvious change in KRN or ear diameter (Fig 6D and S4 and S5 Tables), only *UB3-mum4* showed a slight but significant decrease in KRN in 2013 Wuhan environment (*P*-value = 0.01, Fig 6D and S4 Table). Interestingly, double mutants of *UB3-mum4* and *UB2-mum3* showed a significant decrease in KRN (*P*-value = 2.21 E^-04^) and ear diameter (*P*-value = 2.90 E^-05^) relative to WT (Fig 5D and 5E and S6 Table). In addition, *UB3-mum4* and double mutant also showed a slight but significant reduction in tassel branch number relative to wild types (Fig 6E and S6 Table).

### The potential for use of *KRN4* in maize improvement

The introgression of the 1.2-Kb PAV from NX531 into H21 results in significant enlargement of the inflorescence meristem in the immature ear of H21^NX531^ (Table 1 and Fig 1C and 1D). The enlarged diameter of the inflorescence meristem provides a larger space to support the larger number of spikelet-pair meristems generated. Accompanying the increase in KRN in H21^NX531^, kernel number per ear also significantly increased, but 100-kernel weight was not affected, and so the grain yield of H21^NX531^ was markedly enhanced (Table 1). The enhanced yield resulting from the increased KRN with unaltered kernel weight may only apply to the specific genetic backgrounds or growth conditions. Then, we anticipate that selection for the favorable allele at *KRN4* will contribute positively to maize productivity. To test this hypothesis, we used marker-assisted selection to introgress the *1*.*2-Kb Presence* alleles from two inbred lines carrying the *1*.*2-Kb Presence* alleles, TY6 and Qi205, into W138 and Mo17 carrying the *1*.*2-Kb Absence* alleles. To minimize the influence of genetic background, heterozygotes at the 1.2-Kb PAV in BC_3_F_1_ were selfed to develop a segregating population, and then two homozygous genotype subgroups (*1*.*2-Kb Presence* subgroup, *1*.*2-Kb Absence* subgroup) were identified in each segregating population for KRN evaluation to maximum randomize genetic background. We found that mean of KRN of the *1*.*2-Kb Presence* subgroup was almost 2 rows higher than that of the *1*.*2-Kb Absence* subgroup, indicating that the introgression of the superior alleles could increase KRN of recurrent parents (S7 Table).

## Discussion

### *UB3* is distally regulated by *KRN4* and controls kernel row number in maize

In this study, we fined mapping a major KRN QTL, *KRN4*, and suggested the 3-Kb intergenic region that includes a 1.2-Kb PAV ~60 Kb downstream of *UB3* is the causation underlying the major KRN QTL. Expression analysis in immature ear indicated that the expression difference of *UB3* between H21 and H21^NX531^, and also among diverse inbred lines, was highly correlated with variation in *KRN4*. Further, the weak mutants of *UB3-mum4* and *UB2-mum3* used in this study demonstrated that elevation of *UB3* expression reduces the KRN and ear diameter, which is consistent with previous characterized *ub3* and *ub2* knock-out mutations which cause KRN increase and ear diameter enlargement [13]. The elevation of *UB3* expression in *UB3-mum4*/*UB2-mum3* may reduce the inflorescence meristem size of the developing ear, resulting in formation of less spikelet-paired meristems (SPMs), and then decreased number of kernel rows and ear diameter. This hypothesis can be supported in H21 and H21^NX531^, where the higher *UB3* expression in H21 is correlated with smaller inflorescence meristem size and less SPMs formation than H21^NX531^, and also is consistent with *ub3* knock-out mutants with enlargement in inflorescence meristem size [13]. However, we observed that an increase of *UB3* expression in *UB3-mum4* slightly reduces the tassel branch number, which is inconsistent with the results of *ub3* knock-out mutants, which show highly suppressed tassel branch [13]. These observations imply that the allele effect on tassel branch number of *UB3-mum4* used in this study is different from previous identified *ub3* knock-out mutants. The ortholog of *UB3* and *UB2* in rice, *OsSPL14*, negatively regulates axillary bud outgrowth to repress shoot tillering, but positively regulates the number of panicle branches by enhancing meristematic activity and cell proliferation [19–20, 22–24]. Unlike *OsSPL14*, *UB3* and *UB2* exhibit redundant biological functions on negative regulation of KRN, a kind of short branch in maize ear. It seems like that *UB3* and *UB2* evolved from a common ancestral gene with *OsSPL14* and retained similar biological functions, but may act in opposite ways. Therefore, we suggest that *KRN4* controls the natural variation of KRN by acting as a distal regulator of *UB3* expression and *UB3* negatively regulates KRN in maize.

Previous study revealed that *ub3* shows more severe phenotype than *ub2* [13]. The *UB3* locus is also a KRN and tassel branch number QTLs hotspot detected by many studies [2–3], and *UB3* is found to be the causative gene underlying a major KRN QTL, *KRN4*, in this study. However, the natural variation in *UB2* locus has not been found to be associated with inflorescence traits in maize [2–3]. So, alterations in *UB3* by mutations or natural variation are more likely to cause the response on inflorescence traits than *UB2*. In addition, the expression differences of *UB3* was not in developing tassels, consistent with ear traits being modulated and tassel traits not. Thus, *KRN4* may not be responsible for the TBN QTLs at this locus, which is consistent with previous suggestion that KRN and TBN are controlled by different polymorphisms of *UB3* [13].

The association analysis of *KRN4* revealed that only the 1.2-Kb PAV containing TE fragments was significantly associated with KRN in diverse inbred lines. Hence, variation in KRN between H21 and H21^NX531^ due to *UB3* expression is possibly caused by the 1.2-Kb PAV. This kind of distal regulation of gene expression being responsible for variation in important traits has been previously described in maize, and two different mechanisms may account for it. First, like *tb1*, *Vgt1*, *ZmCCT*, and *prol1*.*1*, the causal sequences (commonly transposon derived sequences) act as enhancers to regulate gene expression level or pattern in cis [18, 25–28]. In a second mechanism, non-coding tandem repeat sequences located ∼100 kb upstream of *b1* express dsRNA, which mediates trans-communication between alleles to establish paramutation [29]. *KRN4* may interact with the *UB3* regulatory region in cis to promote expression of *UB3*, or the transposon fragments in *KRN4* may express small RNAs and affect *UB3* expression by an epigenetic regulation mediated by small RNAs. These assumptions are yet to be investigated.

### *KRN4* and *UB3* might genetically interact to regulate KRN

In addition to 1.2-Kb PAV, an A/G SNP designated as S35 that is significantly associated with KRN was also detected in our association mapping panel. Located in an exon of *UB3*, this is the same as the Ser220Asn polymorphism mentioned by Chuck et al. [13]. S35 showed stronger association with KRN and had better support in conditional analysis than did the 1.2-Kb PAV. However, unlike the 1.2-Kb PAV, in the recombinant lines of the fine mapping population, the introgression of A (or Asn220) from H21^NX531^ to replace the G (or Ser220) in H21 did not result in increased KRN in RL12. Further, when S35 was segregating in RL11-RL12, no significant additive effect was observed. But the additive effects of 1.2-Kb PAV could be promoted 40% by S35 in the background of 1.2-Kb PAV, implying a positive genetic interaction between them and a larger genetic effect due to their combination. This hypothesis is supported by the stronger association of KRN with the creating haplotype 1.2-Kb-PAV-S35 than with either of the individual loci. We propose that a change in UB3 protein function due to S35 made UB3 more efficient in modulating inflorescence development. Although S35 alone or other polymorphisms in linkage disequilibrium with *KRN4* did not display apparent genetic effects in H21, S35 might still affect the biological function of *UB3* in KRN formation in another genetic background. Therefore, the 1.2-Kb-PAV-S35 combination could represent the high- and low-KRN haplotypes for *KRN4* among these diverse inbred lines, and Hap1 was the most favorable haplotype for KRN.

### *KRN4* was a selection target during modern maize domestication and improvement

Domestication leads to the loss of genetic diversity throughout the genome, or in specific regions, and desirable alleles for important traits have been selected and enriched [17–18, 30]. For *KRN4*, the nucleotide diversity in maize landrace is markedly reduced relative to that in teosinte. The strong selection signal was also observed by Tajima’s D test and HKA test. Accompanying the selection on *KRN4*, the *1*.*2-Kb Presence* allele was continuously enriched during maize domestication and improvement for desirable alleles of *KRN4*. Its frequency was increased more than twofold from teosinte to maize landrace, and was further doubled from landrace to modern inbred line. In the corresponding processes, the mean values of allele frequency at four neutral genes (*adh1*, *adh2*, *fus6* and *te1*) were small changed, just 0.37 fold change from teosinte to landrace, and 0.15 fold change from landrace to modern inbred line for low frequent allele, respectively. Additionally, the favourable allele of *KRN4* was enriched rather than was fixed in modern maize lines, which is different from the case of *tga1* ant *tb1*, indicating that *KRN4* may be not the critical locus that determines the transition from 2 rows in teosinte to more than 4 rows in modern maize. This was further supported by the fact that neither *KRN4* nor *UB3* is located within domestication-associated QTL [30]. However, the favourable *A* allele of S35 in *UB3* is not detected in teosinte and has low frequency in maize landraces, indicating that it might have emerged during the post-domestication improvement of modern maize. Because of the larger genetic effect exhibited by the interaction between *1*.*2-Kb Presence* allele of *KRN4* and *A* allele of S35, Hap1 was likely the selection target in modern temperate maize improvement, and the frequency of Hap1 increased more than 7 folds from tropical to temperate maize. Meanwhile, the frequency of *A* allele of S35 is enriched in temperate maize, but the *1*.*2-Kb Presence* allele shows similar frequency between tropical and temperate maize. The decrease of selection pressure on *KRN4* during temperate maize breeding might be caused by the selection on the other KRN loci or the diverse breeding objectives.

Despite the continued improvement during breeding program, the favourable Hap1 is still absent in most modern maize inbred lines that are included in our association mapping panel. For the TST lines in our association mapping panel, Hap1 was still a rare haplotype. Thus *KRN4* and *UB3* could be subjected to more intense selection by molecular breeding to improve yield by increasing number of kernel rows in maize ear. In conclusion, the dissection of *KRN4* in our study not only extends our knowledge about the genetic and molecular mechanisms of important traits in maize, but also provides diagnostic and germplasm tools for improving maize KRN and grain yields.

## Materials and Methods

### Association analysis

A subset of an association mapping panel with 368 diverse inbred lines was genotyped with 500K SNP markers [31]. KRN of these 368 lines was evaluated in five environments and reported in previous study, including Ya'an (30°N, 103°E), Sanya (18°N, 109°E), and Kunming (25°N, 102°E) in 2009, and Wuhan (30°N, 114°E) and Kunming (25°N, 102°E) in 2010 [3]. The best linear unbiased prediction (BLUP) of KRN was estimated using a linear mixed model in SAS software (SAS Institute Inc., 2001) by previous study [3, 32]. The association of *KRN4* with KRN (BLUP data) [3] was established using Tassel v3.0 with a mixed linear model (MLM) approach considering varietal relatedness (K) and population structure (Q) (MLM K + Q) [3, 15–16]. The linkage disequilibrium among associated SNPs was estimated using Haploview v4.1 [33].

### Fine mapping of *KRN4*

A near-isogenic line, H21^NX531^, that incorporates the *KRN4* QTL for kernel row number (Chr4:198.9Mb-199.9Mb, B73 RefGen V2, S1 Fig), was developed by four cycles of backcrossing (BC) followed by two cycles of selfing, using H21 as the recurrent parent and NX531 as the donor of the favorable allele. Over 10,000 F2 individuals derived from the H21×H21^NX531^ cross were genotyped with markers flanking *KRN4* and 14 newly developed markers (Primers were listed in S6 Dataset) within the QTL interval to identify the recombinants. The heterozygous recombinants were self-crossed to segregate the homozygous recombinant (HR) and non-recombinant (HNR) progeny pairs from each recombinant derived family. The HR and NHR progeny pairs were phenotyped at Wuhan (30°N, 114°E) and Sanya (18°N, 109°E) in 2013 (S1 Dataset), with two replications under a randomized block design for each. And the HRs and HNRs were self-crossed to generate homozygous progeny lines for replicated testing at Wuhan and Baoding (38°N, 115°E) in 2014 (S1 Dataset) with two replications under a randomized block design for each. The substitution mapping procedure widely used in fine mapping [34] was employed by examining the KRN differences between HRs and H21, also between HRs and HNRs progeny pairs from each recombinant derived family, using Student’s t-test with significant threshold *P*-value < 0.01.

### Expression analysis

To identify candidate genes for the *KRN4* QTL, analysis of the expression of genes in the relevant interval was performed on developing ears and tassels from H21, H21^NX531^, recombinant lines, and 38 diverse inbred maize lines (S1 Table) using Quantitative PCR (qPCR). Total RNA was extracted using TRIzol Reagent (Life Technologies, Invitrogen, Carlsbad, CA, USA). Total RNAs of H21 and H21^NX531^ lines were extracted from roots, leaves, stems, immature 5-mm tassel (5-mm tassel, 6-leaf stage with branch meristem initiation), immature 10-mm tassel (10-mm tassel, 10-leaf stage with branches), immature ear stage 1 (2-mm ear, 10-leaf stage with Inflorescence meristems IMs and spikelet-pair meristems SPMs), and immature ear stage 2 (5-mm ear, 12-leaf stage with IM, SPM, and spikelet-meristems SM). Total RNAs of 38 diverse maize inbred lines were extracted from immature ears at the S1 stage (S1 Table). Total RNAs of *UB3-mum4* and *UB2-mum3* lines were extracted from immature 5-mm tassel and 2-mm ear, respectively. DNase I (TaKaRa Biotech, Dalian, China) was used to remove genomic DNA contamination. An oligo(dT) primer and M-MLV reverse transcriptase (Invitrogen, Carlsbad, CA, USA) were used to synthesize first-strand cDNAs. A SYBR Green RT-PCR kit (Bio-Rad, Hercules CA, USA) was used to perform qPCR with gene-specific primers (S7 Dataset). Expression levels were normalized using beta-actin (NM_001155179) as an endogenous control. The expression data for *UB3* and GRMZM2G001541 in B73 were downloaded from the qTeller website (www.qteller.com) and MaizeGDB website (www.maizegdb.org).

### Mutant analysis

Two *Mutator*-mediated insertion mutants were obtained from the Maize Genetics Cooperation Stock Center at the University of Illinois, Champaign-Urbana. According to information from the Maize Stock Center [35], *UB3-mum4* (UFMu-06293) has *Mutator* (*Mu*) inserted upstream of *UB3*, and *UB2-mum3* (UFMu-06514) has *Mu* inserted into the first intron of *UB2*. The insertion site of *Mu* was detected by PCR with gene-specific primers and TIR6 primers designed from the TIR sequence of *Mu* (S3 Dataset). To characterize the phenotypic effects of the mutants and eliminate the influence of the other Mu insertion, *UB3-mum4* and *UB2-mum3* were backcrossed with its parent W22, and self-crossed to develop the F_2_ segregating populations. In each segregating population, wild types (+/+) and homozygous mutants (-/-) were identified by genotyping (Primers used are listed in S3 Dataset) and were phenotyped. The *UB3-mum4* and *UB2-mum3* were crossed to develop double mutant, which was also crossed with W22, and self-crossed to develop F_2_ segregating populations. In the segregating populations, double mutant and wild type individuals were genotyped and phenotyped. Student's t-test was used to evaluate the phenotypic differences between wild types and mutants.

### Analysis of nucleotide diversity and molecular evolution

To discover DNA sequence variation and putative causal polymorphisms in *UB3* and *KRN4*, gene-specific primers (S3 Dataset) were designed to amplify *UB3* and *KRN4* in the association mapping panel [3, 14]. We genotyped 428 inbred lines using the 1.2-Kb PAV in *KRN4* as a marker, and sequenced about 4.0 Kb of DNA from 5'-upstream of *UB3* to its 3'-UTR and ~3 Kb containing *KRN4* in 110 or 428 inbred lines of the AM panel, respectively (the line number of the lines that were sequenced is listed in the S3 Dataset). SNPs and indels with MAF > 0.05 were used to estimate pairwise LD and to evaluate the association between polymorphic sites and KRN under the MLM K+ Q model [15–16]. Conditional analysis was conducted using the associated sites as covariates under an MLM K + Q model in Tassel v3.0. The MLM K + Q model was also used for haplotype-based association analysis. The selfed progeny of heterozygous recombinants RL6-RL12 which are segregating at 1.2-Kb PAV, S35 and S23 were employed to evaluate the genetic effect of 1.2-Kb PAV, S35 and S23 (S1 Dataset). The individuals, which harbored homozygous alleles of the three sites, were used to estimate the additive effects of in each segregating population (S1 Dataset).

The selection pressure on *KRN4* during the domestication and improvement of maize was estimated using 36 randomly selected landraces (S4 Dataset) from 280 diverse maize landrace collections (S5 Dataset) [36] and 29 *Z*. *mays* subsp. *parviglumis* teosinte (S4 Dataset) from 120 teosinte accessions (S5 Dataset). The *KRN4* genomic region was amplified and sequenced using primers listed in S3 Dataset. Nucleotide diversity (π) and Tajima’s D were estimated using DnaSP ver. 5.0 [37]. The 1.2-Kb PAV was treated as single PAV when estimating the nucleotide diversity (π). Four neutral loci (*adh1*, *adh2*, *fus6* and *te1*) [38–41] were used as controls for the HKA test [42] using *Zea diploperennis* as the outgroup. The overall HKA *P*-value was obtained by summing the individual χ2 values of the four control genes. Another 88 teosinte accessions (including 35 *Z*. *mays* subsp. *parviglumis* and 54 *Z*. *mays* subsp. *mexicana* accessions, S5 Dataset) and 244 maize landraces were genotyped by a PCR marker for the 1.2-Kb PAV and a KASP marker (http://www.kbioscience.co.uk/) for S35, to estimate their frequency in teosinte accessions and maize landraces (Primers are listed in S3 Dataset). All of the sequences have been deposited in NCBI Genebank KT928654—KT931615.

### Phylogenetic tree of SBP-box proteins in six plant species

A total of 130 SBP-box genes were predicted in six plant species, including 16 SBP-box genes from *Arabidopsis*, 18 from *Brachypodium*, 18 from sorghum, 19 from rice, 20 from foxtail millet, and 29 from maize [43–48], and used for phylogenetic analysis.

## Supporting Information

S1 Fig Manhattan Plot of the *KRN4* chromosome region. An LD heatmap was constructed using pairwise R2 of the nine KRN-associated SNPs in 368 inbred lines. The X axis represents genomic locations of SNP and Y axis represents -log10(P-observed). The three red points indicate the SNPs most highly associated with KRN, and the dotted line indicates a SNP located in *UB3*. The horizontal lines represent–log10(0.05/N) and–log10(1/N).
(TIF)

S2 Fig Expression patterns of *UB3* (GRMZM2G460544) and GRMZM2G001541 in various tissues of B73. The expression data is obtained from qTeller (www.qteller.com) and MaizeGDB (www.maizegdb.org). Expression pattern of *UB3* observed from qteller (A) and MaizeGDB (B). Expression pattern of GRMZM2G001541 observed from qteller (C) and MaizeGDB (D).
(TIF)

S3 Fig Expression profiling of *UB3* in various tissues of H21 and H21^NX531^, and correlation between expression of *UB3* and *KRN* in these 38 inbred lines. A) 5-mm tassel: 6-leaf stage, with BM initiating; 10-mm tassel: 10-leaf stage, with BM; 2-mm ear: 10-leaf stage, with IM and SPM; 5-mm ear: 12-leaf stage, with IM, SPM, and SM. B) The correlation between expression of *UB3* and KRN in these 38 inbred lines.
(TIF)

S4 Fig Pattern of pairwise linkage disequilibrium in *UB3* and *KRN4* region. All polymorphisms with a minor allele frequency (MAF) >5% were used to calculate the pairwise linkage disequilibrium (LD). The four polymorphisms most significantly associated with KRN are indicated. In the gene structure of *UB3*, the blue boxes represent the transposon fragments inserted in the promoter region (S23), the white boxes represent the UTR regions, the black boxes and the yellow boxes represent exons, and the yellow boxes also represent the SBP-box domain.
(TIF)

S5 Fig Phylogenetic tree of SBP-box proteins in six plant species. The legend indicates the scale of branch lengths. Different colors represent the 14 different subfamilies of SBP-box genes.
(PDF)

S6 Fig The insertion site of *Mutator* in *UB2-mum3* and its alternative spliced transcripts in *UB2-mum3*. A) Detection of alternative spliced transcripts of *UB2* in *UB2-mum3*. The *Mu*IS-Primer (S7 Dataset) was used to amplify the cDNA sequence of *UB2* flanking the *Mu7* insertion site. In the 2-mm ear sample of *UB2-mum3*, a larger band than the predicted transcript was observed. B) A diagram of the sequence composition of the alternatively spliced transcript of *UB2*. A 145-bp segment originating from the intron flanking the *Mu7* insertion site and a 150-bp segment consist of the terminal *Mu7* inverted repeat.
(TIF)

S1 Table The 38 maize inbred lines used for expression analysis. (DOCX)

S2 Table Conditional association analysis of the four associated sites in *KNR4* and *UB3*. (DOCX)

S3 Table Input values used to perform the HKA tests. (DOCX)

S4 Table Phenotypic variation in the *UB3-mum4* mutant and wild type at Wuhan and Sanya in 2013. (DOC)

S5 Table Phenotypic variation in *UB2-mum3* mutant and wild type at Sanya in 2013. (DOC)

S6 Table Phenotypic variation in *UB3-mum4* and *UB2-mum3* double mutants and wild type at Wuhan in 2014. (DOCX)

S7 Table Marker-assisted selection to test the genetic effects of Hap1 in BC_3_F_2_. (DOC)

S1 Dataset Progeny test of the 13 recombinants. (XLS)

S2 Dataset Sequence comparison of the ~3 Kb region of *KRN4* between H21 and NIL (H21^NX531^). (PDF)

S3 Dataset The primer sequences used for genotyping mutant and re-sequencing maize association mapping panel, landrace and teosinte. (XLS)

S4 Dataset List of teosinte collections and maize landraces used in nucleotide diversity analysis. (XLS)

S5 Dataset List of teosinte accessions and maize landraces used for detecting frequencies S23 and 1.2-Kb PAV. (XLS)

S6 Dataset The primer sequences used for genotyping recombination lines. (XLS)

S7 Dataset All primers used for expression analysis. (XLS)
