# Supplementary figures and images for: KRN4 Controls Quantitative Variation in Maize Kernel Row Number
Source: PLoS Genet. 2015 Nov 17;11(11):e1005670. doi: 10.1371/journal.pgen.1005670 (PMC4648495; doi:10.1371/journal.pgen.1005670)

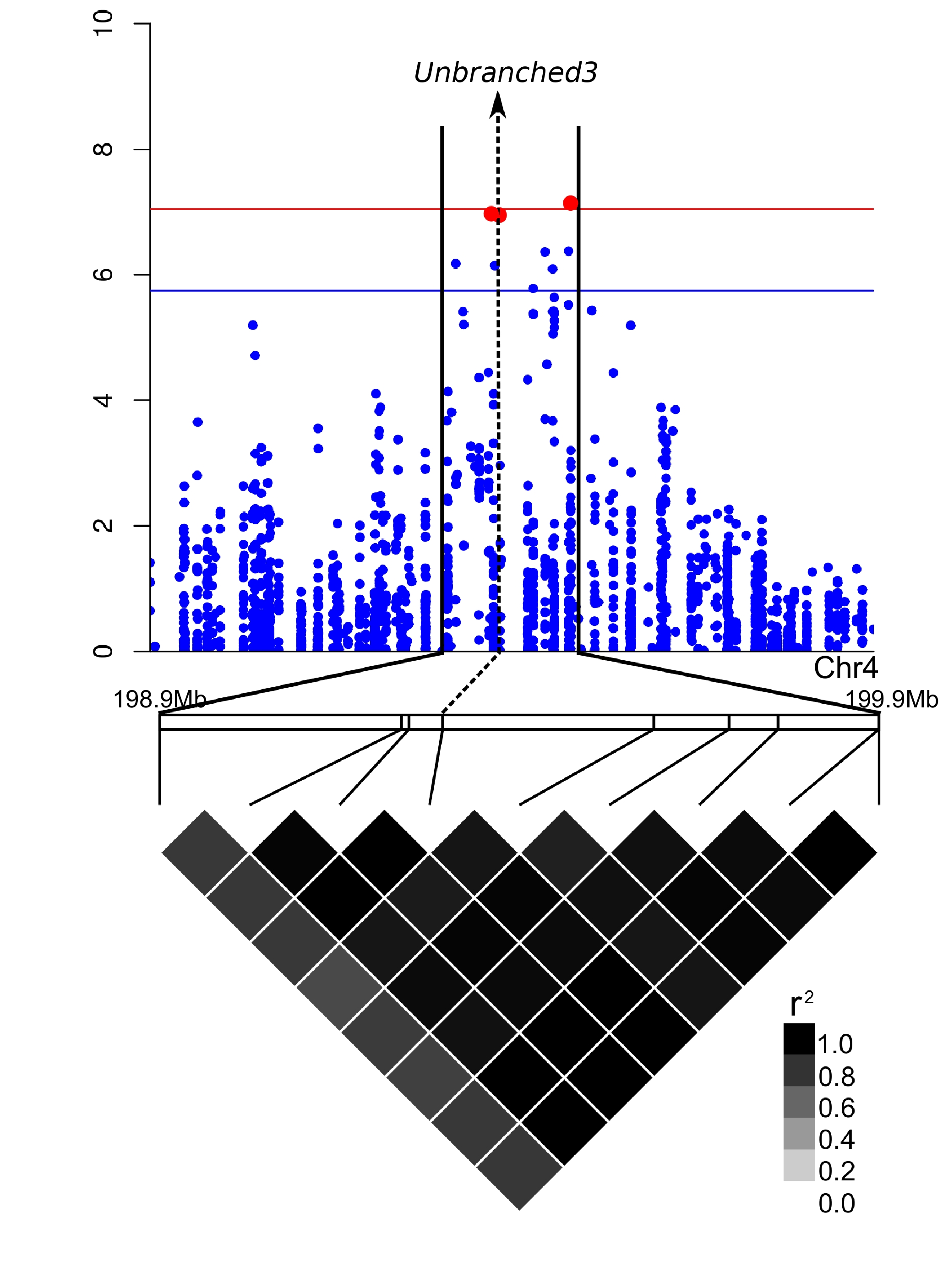

Supplement: S1 Fig — An LD heatmap was constructed using pairwise R2 of the nine KRN-associated SNPs in 368 inbred lines. The X axis represents genomic locations of SNP and Y axis represents -log10(P-observed). The three red points indicate the SNPs most highly associated with KRN, and the dotted line indicates a SNP located in UB3. The horizontal lines represent–log10(0.05/N) and–log10(1/N). (TIF) [file pgen.1005670.s001.tif]

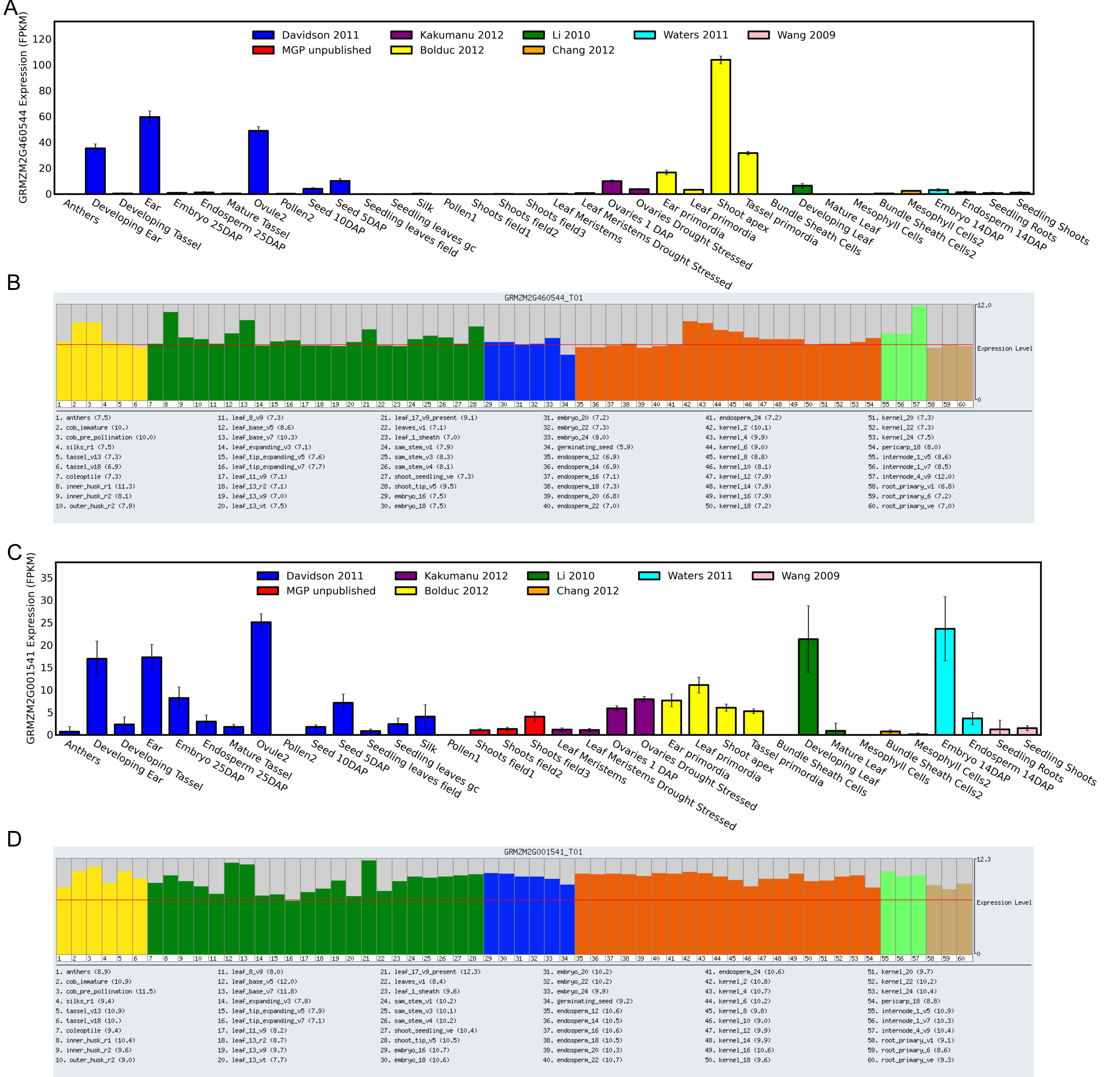

Supplement: S2 Fig — The expression data is obtained from qTeller (www.qteller.com) and MaizeGDB (www.maizegdb.org). Expression pattern of UB3 observed from qteller (A) and MaizeGDB (B). Expression pattern of GRMZM2G001541 observed from qteller (C) and MaizeGDB (D). (TIF) [file pgen.1005670.s002.tif]

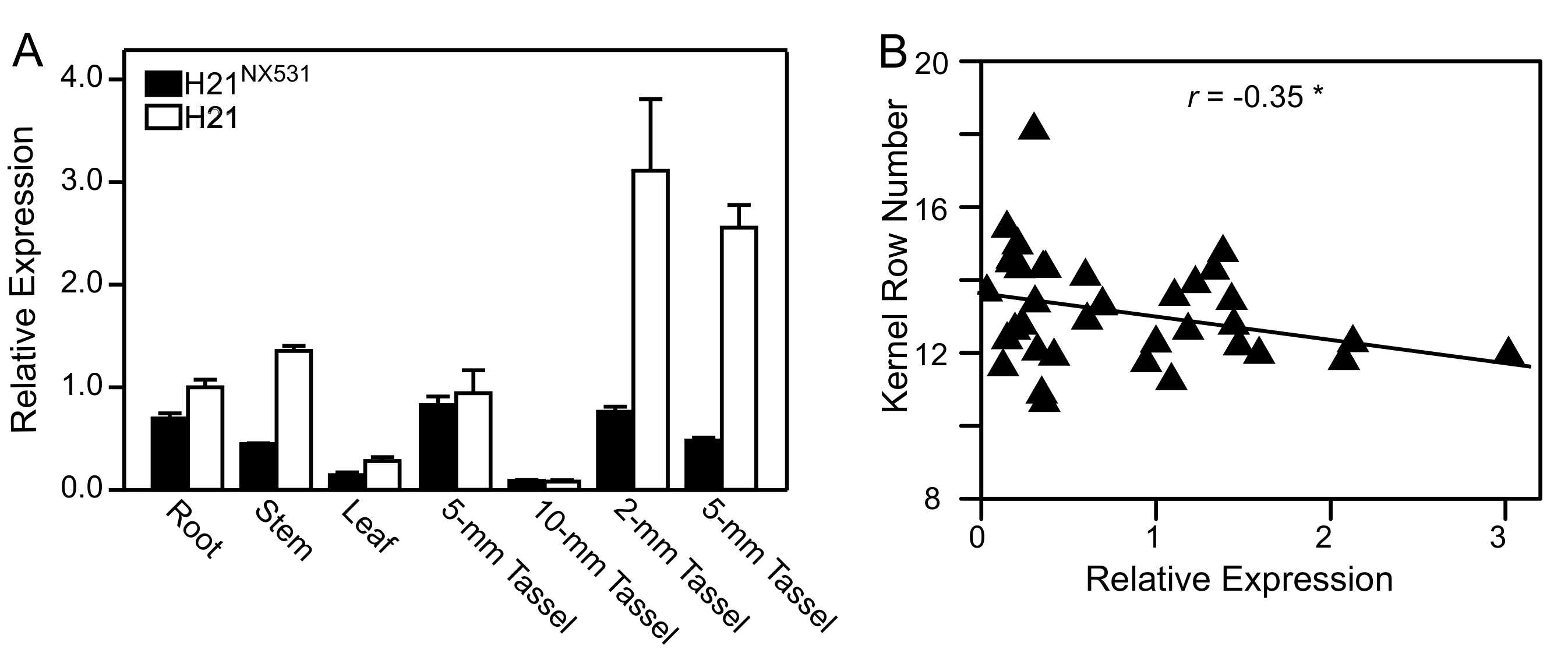

Supplement: S3 Fig — A) 5-mm tassel: 6-leaf stage, with BM initiating; 10-mm tassel: 10-leaf stage, with BM; 2-mm ear: 10-leaf stage, with IM and SPM; 5-mm ear: 12-leaf stage, with IM, SPM, and SM. B) The correlation between expression of UB3 and KRN in these 38 inbred lines. (TIF) [file pgen.1005670.s003.tif]

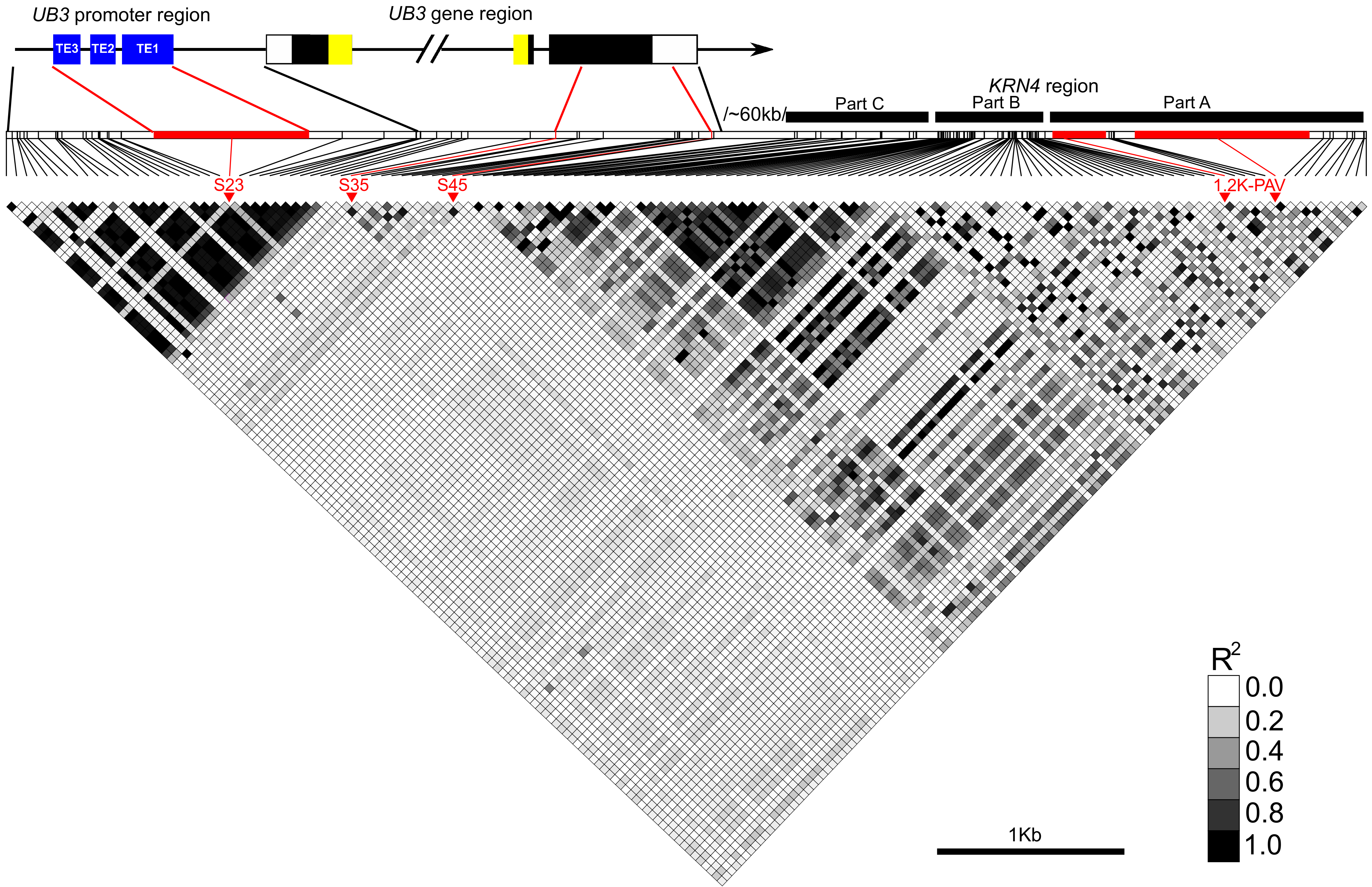

Supplement: S4 Fig — All polymorphisms with a minor allele frequency (MAF) >5% were used to calculate the pairwise linkage disequilibrium (LD). The four polymorphisms most significantly associated with KRN are indicated. In the gene structure of UB3, the blue boxes represent the transposon fragments inserted in the promoter region (S23), the white boxes represent the UTR regions, the black boxes and the yellow boxes represent exons, and the yellow boxes also represent the SBP-box domain. (TIF) [file pgen.1005670.s004.tif]

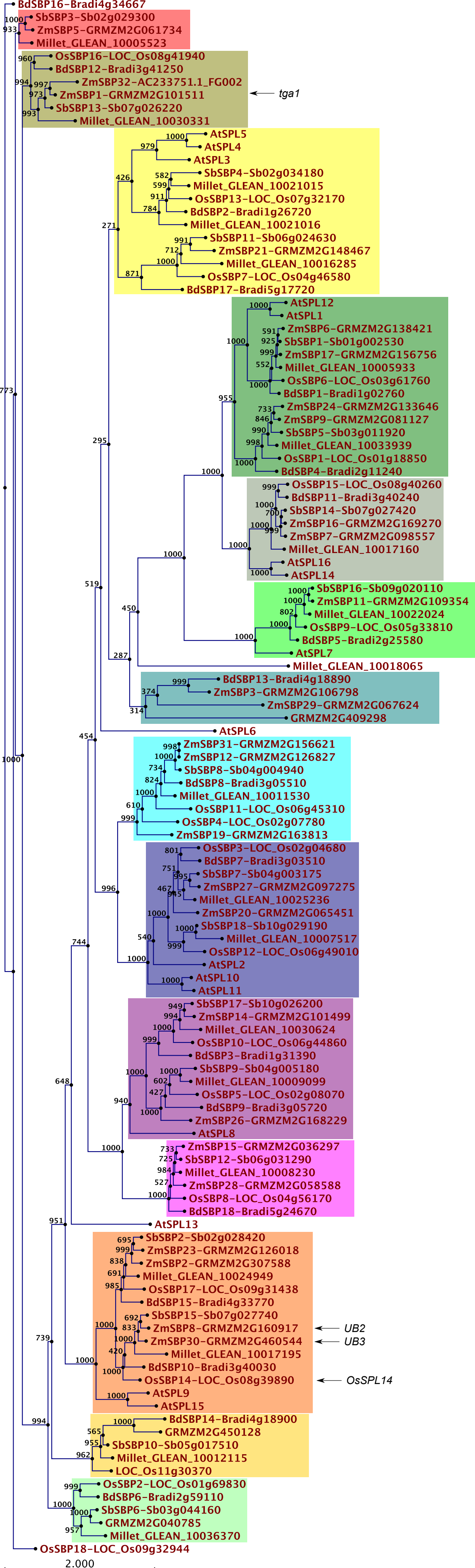

Supplement: S5 Fig — The legend indicates the scale of branch lengths. Different colors represent the 14 different subfamilies of SBP-box genes. (PDF) [file pgen.1005670.s005.pdf]

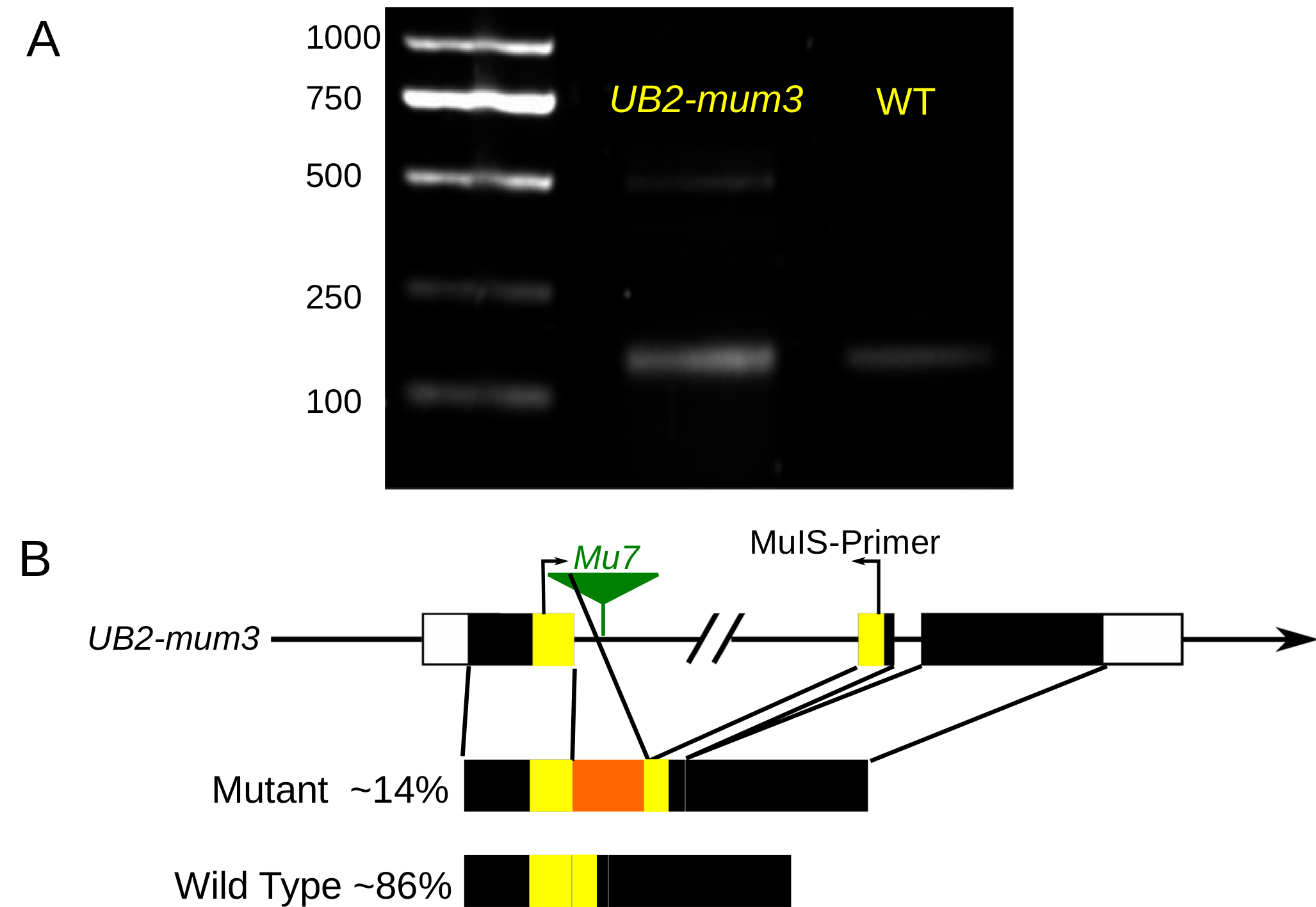

Supplement: S6 Fig — A) Detection of alternative spliced transcripts of UB2 in UB2-mum3. The MuIS-Primer (S7 Dataset) was used to amplify the cDNA sequence of UB2 flanking the Mu7 insertion site. In the 2-mm ear sample of UB2-mum3, a larger band than the predicted transcript was observed. B) A diagram of the sequence composition of the alternatively spliced transcript of UB2. A 145-bp segment originating from the intron flanking the Mu7 insertion site and a 150-bp segment consist of the terminal Mu7 inverted repeat. (TIF) [file pgen.1005670.s006.tif]

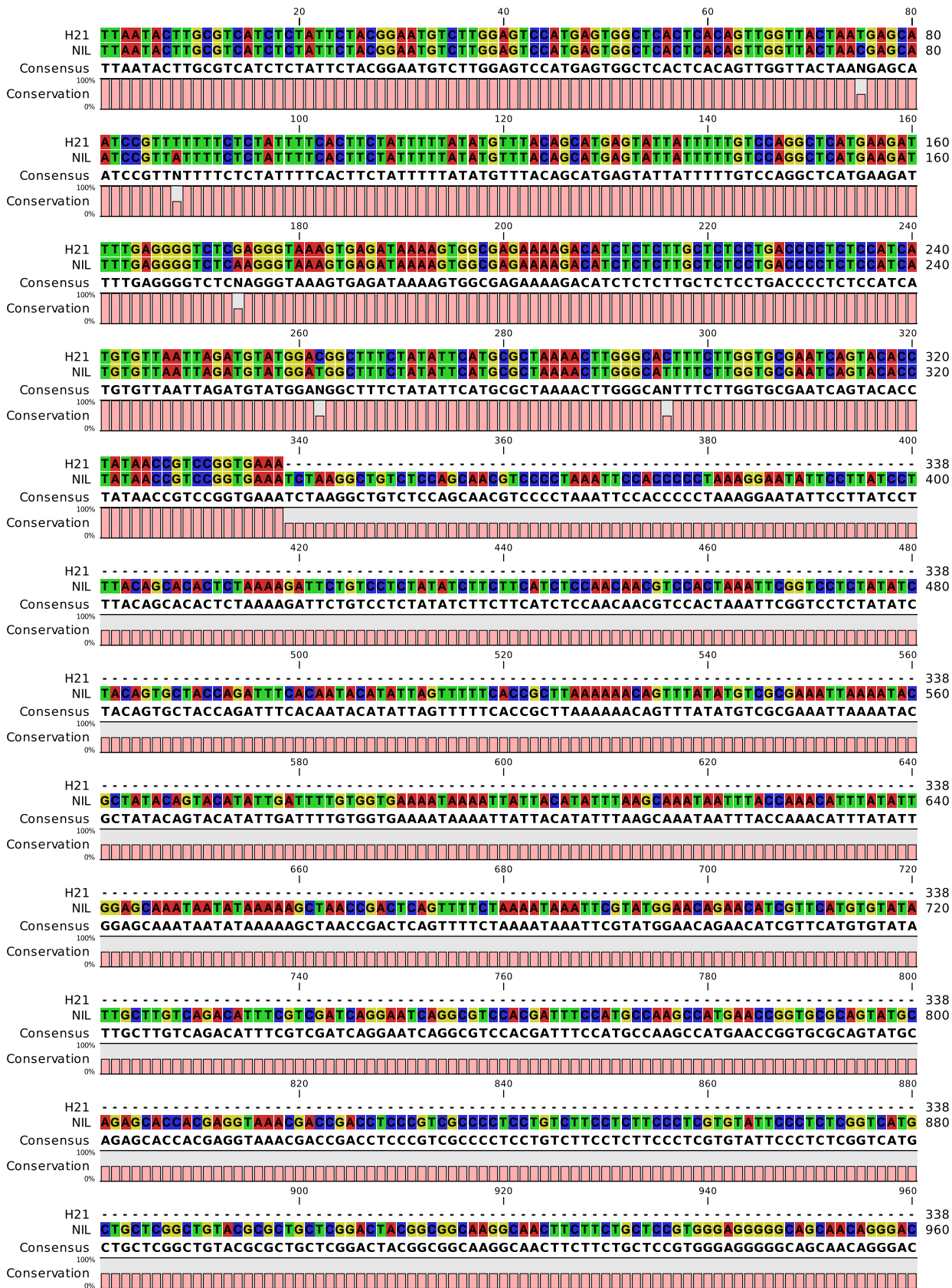

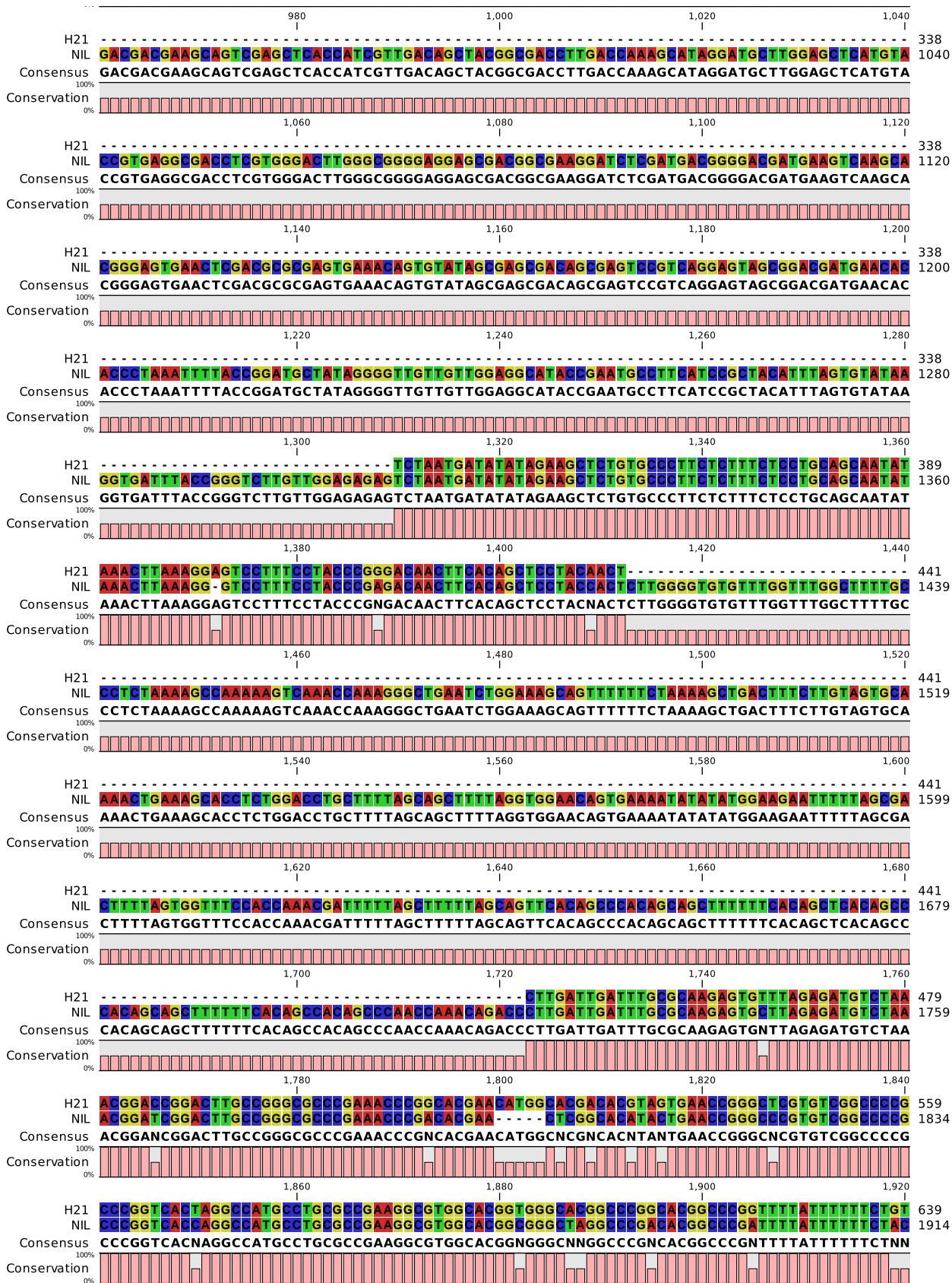



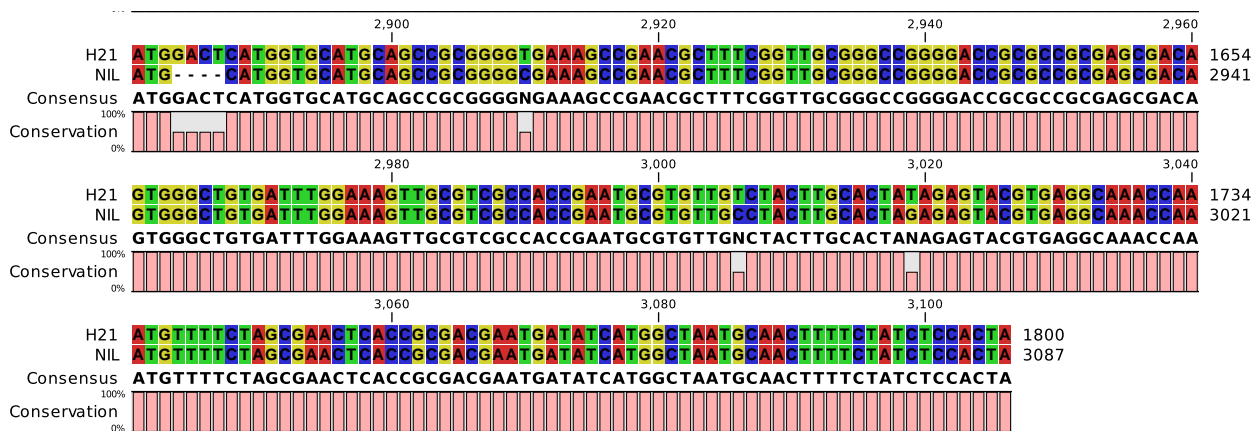

Supplement: S2 Dataset — (PDF) [file pgen.1005670.s015.pdf]
