# Supplementary material for: KRN4 Controls Quantitative Variation in Maize Kernel Row Number
Source: PLoS Genet. 2015 Nov 17;11(11):e1005670. doi: 10.1371/journal.pgen.1005670 (PMC4648495; doi:10.1371/journal.pgen.1005670)
Supplement: S1 Table — (DOCX) [file pgen.1005670.s007.docx]

**S1 Table. The 38 maize inbred lines used for expression analysis**

| Line name | Genotype at *KRN4* | Group^*^ | Kernel row number |
| --- | --- | --- | --- |
| HUANGC | 1.2-Kb insertion | H | 14.31 |
| 1462 | 1.2-Kb insertion | H | 14.75 |
| CIMBL71 | 1.2-Kb insertion | H | 14.57 |
| 05WN230 | 1.2-Kb insertion | H | 13.55 |
| LIAO159 | 1.2-Kb insertion | H | 14.32 |
| HB | 1.2-Kb insertion | H | 10.87 |
| D047 | 1.2-Kb insertion | H | 11.62 |
| B11 | 1.2-Kb insertion | H | 12.62 |
| YE52106 | 1.2-Kb insertion | H | 13.33 |
| JIAO51 | 1.2-Kb insertion | H | 15.42 |
| TY8 | 1.2-Kb insertion | H | 13.67 |
| B73 | 1.2-Kb insertion | H | 14.32 |
| CIMBL123 | None | L | 11.96 |
| LY042 | None | L | 11.24 |
| CIMBL123 | None | L | 11.96 |
| CIMBL121 | None | L | 12.05 |
| CIMBL95 | None | L | 12.76 |
| 238 | None | L | 14.27 |
| CIMBL95 | None | L | 12.76 |
| BY4960 | None | L | 13.29 |
| K12 | None | L | 12.90 |
| DAN598 | None | L | 18.11 |
| LY | None | L | 12.28 |
| D863F | None | L | 13.90 |
| CIMBL157 | None | L | 12.19 |
| ZHENG653 | None | L | 14.96 |
| CIMBL89 | None | L | 11.91 |
| BY815 | None | L | 12.62 |
| MO17 | None | L | 10.64 |
| MN | None | L | 13.37 |
| BY855 | None | L | 14.47 |
| YE107 | None | L | 12.36 |
| SHEN137 | None | L | 11.73 |
| DSB | None | L | 11.79 |
| U8112 | None | L | 13.44 |
| 150 | None | L | 14.10 |
| 18-599 | None | L | 14.57 |
| GEMS2 | None | L | 12.27 |

^*^H represents the line holding *KRN4^NX531^* allele, L represents the line holding *KRN4^H21^* allele. All of the maize lines were public available in http://www.maizego.org.
