# Supplementary material for: KRN4 Controls Quantitative Variation in Maize Kernel Row Number
Source: PLoS Genet. 2015 Nov 17;11(11):e1005670. doi: 10.1371/journal.pgen.1005670 (PMC4648495; doi:10.1371/journal.pgen.1005670)
Supplement: S2 Table — (DOCX) [file pgen.1005670.s008.docx]

**S2 Table. Conditional association analysis of the four associated sites in *KNR4* and *UB3*.**

| Condition | S35 | S23 | S45 | 1.2-Kb PAV |
| --- | --- | --- | --- | --- |
| S35 | NA | 0.41 | 0.49 | 0.03 |
| S23 | 4.44E-07 | NA | 0.25 | 2.85E-05 |
| S45 | 4.82E-05 | 1.52E-03 | NA | 2.18E-05 |
| 1.2-Kb PAV | 5.76E-05 | 4.02E-03 | 3.39E-03 | NA |

The observed P-value was estimated by MLM+Q model with the condition site as covariate.
