# Supplementary material for: KRN4 Controls Quantitative Variation in Maize Kernel Row Number
Source: PLoS Genet. 2015 Nov 17;11(11):e1005670. doi: 10.1371/journal.pgen.1005670 (PMC4648495; doi:10.1371/journal.pgen.1005670)
Supplement: S3 Table — (DOCX) [file pgen.1005670.s009.docx]

S3_Table. Input values used to perform the HKA tests

| Loci^a^ | S^b^ | L^c^ | N^d^ | K^e^ |
| --- | --- | --- | --- | --- |
| *Adh1* | 41 | 684 | 39 | 5.36842 |
| *Adh2* | 30 | 562 | 30 | 7.0046 |
| *Te1* | 13 | 813 | 28 | 1.07389 |
| *Fus6* | 28 | 378 | 51 | 2.33412 |
| *KRN4* | 63 | 1722 | 55 | 5.08013 |

^a^ Four neutral genes and ~3-Kb genomic sequence of *KRN4*(Fig. 5A).

^b^ Number of segregating sites.

^c^ Number of total sites excluding gaps.

^d^ Sample size.

^e^ Average nucleotide difference.
