# Supplementary material for: KRN4 Controls Quantitative Variation in Maize Kernel Row Number
Source: PLoS Genet. 2015 Nov 17;11(11):e1005670. doi: 10.1371/journal.pgen.1005670 (PMC4648495; doi:10.1371/journal.pgen.1005670)
Supplement: S4 Table — (DOC) [file pgen.1005670.s010.doc]

**S4 Table. Phenotypic variation in *UB3-mum4* mutant and wild type at Wuhan and Sanya in 2013**

|  | 2013 Wuhan | | | |  | 2013 Sanya | | | |
| --- | --- | --- | --- | --- | --- | --- | --- | --- | --- |
| *UB3* | - | + | P-value | N |  | - | + | P-value | N |
| *UB2* | + | + |  |  |  | + | + |  |  |
| KRN | 15.8 ± 1.1 | 16.4 ± 0.9 | 0.01 | 49/39 |  | 14.8 ± 0.7 | 14.9 ± 0.6 | 0.6 | 18/24 |
| Total BN | 15.5 ± 3.6 | 18.7 ± 3.5 | 9.4 E^-06^ | 48/41 |  | 7.2 ± 1.3 | 8.4 ± 1.7 | 0.01 | 25/21 |
| Primary BN | 12.3 ± 3.4 | 14.1 ± 3.4 | 0.01 | 48/41 |  | 6.5 ± 1.0 | 7.0 ± 0.9 | 0.10 | 25/21 |
| Secondary BN | 3.2 ± 1.1 | 4.7 ± 1.5 | 1.6 E^-08^ | 48/41 |  | 0.7 ± 0.5 | 1.4 ± 0.9 | 1.70 E^-03^ | 25/21 |
| ED (mm) | 31.5 ± 2.0 | 32.1 ± 2.0 | 0.37 | 23/17 |  | 39.4 ± 1.4 | 37.9 ± 1.6 | 0.85 | 18/24 |
| EL (cm) | 13.2 ± 1.2 | 13.5 ± 1.3 | 0.58 | 25/17 |  | 13.5 ± 0.5 | 14.6 ± 0.6 | 4.56 E^-05^ | 18/24 |
| KNR | 30.4 ± 2.6 | 30.8 ± 2.8 | 0.64 | 25/17 |  | 27.3 ± 1.8 | 27.3 ± 1.4 | 0.99 | 16/24 |

KRN: kernel row number; Total BN: Total branch number; Primary BN: Primary branch number; Secondary BN: Secondary branch number;

ED: ear diameter; EL: ear length; KNR: kernel number per row;

N: sample size, mutant/wild type;Mean ± SD.
