# Supplementary material for: KRN4 Controls Quantitative Variation in Maize Kernel Row Number
Source: PLoS Genet. 2015 Nov 17;11(11):e1005670. doi: 10.1371/journal.pgen.1005670 (PMC4648495; doi:10.1371/journal.pgen.1005670)
Supplement: S5 Table — (DOC) [file pgen.1005670.s011.doc]

**S5 Table. Phenotypic variation in *UB2-mum3* mutant and wild type at Sanya in 2013.**

|  | Sanya 2013 | | | |
| --- | --- | --- | --- | --- |
| *UB3* | **+** | + | P-value | N |
| *UB2* | - | + |
| KRN | 14.6 ± 1.8 | 14.9 ± 1.2 | 0.51 | 21/27 |
| Total BN | 7.9 ± 2.5 | 8.1 ± 2.1 | 0.70 | 21/26 |
| Primary BN | 6.7 ± 2.5 | 6.8 ± 2.0 | 0.89 | 21/26 |
| Secondary BN | 1.2 ± 1.0 | 1.4 ± 1.0 | 0.59 | 20/25 |
| ED (mm) | 40.8 ± 5.0 | 38.9 ± 3.0 | 0.13 | 20/23 |
| EL (cm) | 14.0 ± 1.7 | 13.9 ± 1.4 | 0.82 | 20/23 |
| KNR | 27.4 ± 3.8 | 27.7 ± 2.5 | 0.71 | 19/23 |

KRN: kernel row number; Total BN: Total branch number; Primary BN: Primary branch number; Secondary BN: Secondary branch number; ED: ear diamter; EL: ear length; KNR: kernel number per row; N: sample size, mutant/wild type. NA: Not analyzed; Mean ± SD.
