# Supplementary material for: KRN4 Controls Quantitative Variation in Maize Kernel Row Number
Source: PLoS Genet. 2015 Nov 17;11(11):e1005670. doi: 10.1371/journal.pgen.1005670 (PMC4648495; doi:10.1371/journal.pgen.1005670)
Supplement: S6 Table — (DOCX) [file pgen.1005670.s012.docx]

S6 Table. Phenotypic variation in *UB3-mum4* and *UB2-mum3* double mutants and wild type in Wuhan in 2014

| Traits | *UB3-mum4*  *UB2-mum3* | Wild Type | P-value | N |
| --- | --- | --- | --- | --- |
| KRN | 14.1 ± 1.6 | 16.0 ± 1.4 | 2.21E^-04^ | 21/22 |
| ED(mm) | 33.2 ± 1.8 | 35.8 ± 1.8 | 2.90E^-05^ | 21/22 |
| EL(cm) | 14.1 ± 1 | 14.8 ± 1.3 | 0.07 | 21/22 |
| KNR | 28.5 ± 2.2 | 30.9 ± 3.7 | 0.01 | 21/22 |
| Total BN | 6.7 ± 1.6 | 9.0 ± 1.9 | 1.13E^-04^ | 21/22 |
| Primary BN | 5.9 ± 1.3 | 7.6 ± 1.4 | 2.50E^-04^ | 21/22 |
| Secondary BN | 0.9 ± 0.7 | 1.5 ± 1.1 | 0.04 | 21/22 |

KRN: kernel row number; Total BN: Total branch number; Primary BN: Primary branch number; Secondary BN: Secondary branch number; ED: ear diamter; EL: ear length; KNR: kernel number per row; N: sample size, mutant/wild type; Mean ± SD
