# Supplementary material for: KRN4 Controls Quantitative Variation in Maize Kernel Row Number
Source: PLoS Genet. 2015 Nov 17;11(11):e1005670. doi: 10.1371/journal.pgen.1005670 (PMC4648495; doi:10.1371/journal.pgen.1005670)
Supplement: S7 Table — (DOC) [file pgen.1005670.s013.doc]

**S7 Table. Marker-assisted selection to test the genetic effects of *KRN4* in BC_3_F_2_**

| Population | 1.2-Kb presence^a^ | None^b^ | P-value^c^ | N^d^ |
| --- | --- | --- | --- | --- |
| W138 × Qi205 | 12.1 ±1.6 | 10.4 ±1.2 | 4.53 E^-04^ | 18/23 |
| W138 × TY6 | 12.2 ±1.3 | 10.5 ±1.0 | 4.83 E^-07^ | 26/34 |
| Mo17 × Qi205 | 11.1 ±1.2 | 10.4 ±0.8 | 0.03 | 45/19 |
| Mo17 × TY6 | 12.2 ±1.3 | 11.0 ±1.2 | 4.34 E^-04^ | 56/25 |

^ab^ 1.2-Kb presence/None represented the genotyopes of the 1.2-Kb PAV in BC_3_F_2_ lines; ^c^ The P-values were observed by Student’s t-test. ^d^ N represents sample size.
